# Kinesin-5 and kinesin-14 are partially antagonistic in spindle assembly in the holocentric silkworm *B. mori* cells

**DOI:** 10.17912/micropub.biology.000630

**Published:** 2022-08-22

**Authors:** Inaara T. Kassamaly, Gaetan Cornilleau, Ines A. Drinnenberg, Phong T. Tran

**Affiliations:** 1 Institut Curie, PSL Université, Sorbonne Université, CNRS, Paris, France; 2 Université de Bordeaux, International Master Program in Cancer Biology, Bordeaux, France; 3 University of Pennsylvania, Department of Cell and Developmental Biology, Philadelphia, PA, United States

## Abstract

We previously showed that the silkworm holocentric spindles are square-shaped, compared to the canonical oval shape of human monocentric spindles (Vanpoperinghe et al. 2021). Further, while kinesin-5 depletion resulted in monopolar spindles in both cells, kinesin-14 depletion affected only the silkworm cells, resulting in mal-shaped spindles (Vanpoperinghe et al. 2021). We now extend our study to quantify the effect of kinesin-5 and kinesin-14 on spindle assembly dynamics and chromosome segregation in holocentric silkworm BmN4 cells. We find that mal-shaped spindle and prolonged mitosis duration are highly correlated with chromosome segregation error, leading to aneuploidy and cell death in BmN4 cells. Further, double RNAi-mediated depletion of kinesin-5 and kinesin-14 partially rescue the monopolar spindle and mal-shaped spindle phenotypes in kinesin-5 and kinesin 14-depleted cells, respectively.

**Figure 1. Kinesin-5 and kinesin-14 organize the spindle in holocentric silkworm BmN4 cells:**
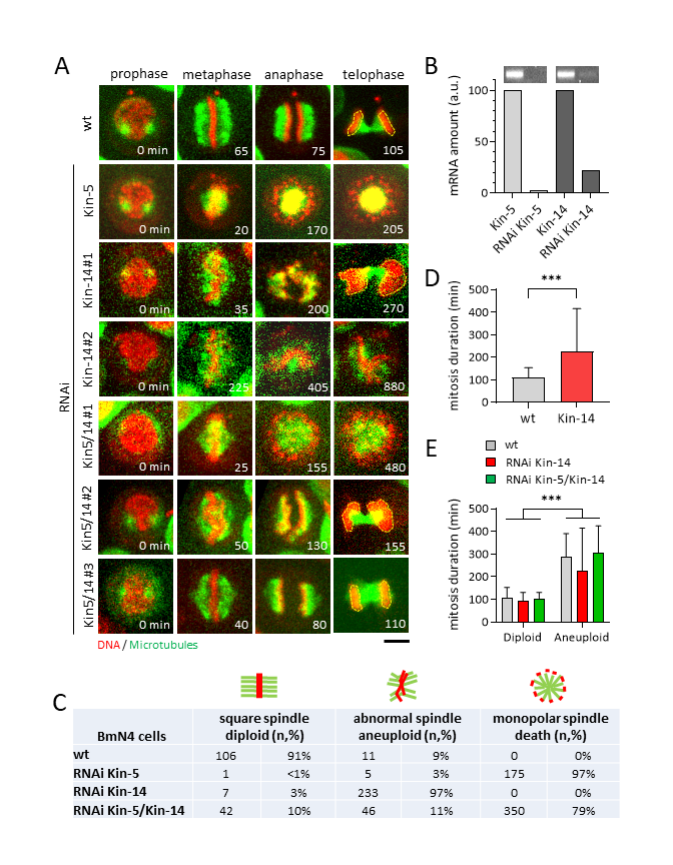
**A.** Time-stamped images of BmN4 cells progressing through mitosis. Dashed yellow lines outline the chromosome mass/area at telophase. Equal DNA mass/area is considered diploid, and unequal DNA mass/area is considered aneuploid. Control (wt) cells showed predominant “square” spindles with compact “straight” chromatids at metaphase, with diploid DNA at telophase. RNAi-mediated depletion of kinesin-5 resulted in monopolar spindles, and subsequent cell death. Depletion of kinesin-14 revealed two phenotypes: abnormal spindle shape, curved dynamic chromatids, prolonged mitosis duration, and aneuploid chromosome segregation (Kin-14#1, 68% of cells); and sustained abnormal spindle and chromosome dynamics that failed to divide during 20 hr of observation (Kin-14#2, 32% of cells). Double-depletion of kinesin-5 and kinesin-14 revealed three phenotypes: monopolar spindles (Kin5/14#1, 79% of cells); abnormal spindle shape and curved dynamic chromatids (Kin5/14#2, 11% of cells; and relatively square-shaped spindles, straight compact chromatids, and diploid chromosome segregation (Kin5/14#3, 10% of cells). Scale bar, 10 µm. **B.** Plot of mRNA level in RNAi-mediated depletion. **C.** Table quantifies (n = number of cells, % = percentage in observed population) the three observed spindle and chromosome segregation phenotypes. **D.** Plot of mitosis duration for cells which completed mitosis during the 20 hr observation time. Control (wt) cells: 109 ± 45 min (n=64); RNAi kinesin-14 (Kin-14): 226 ± 191 min (n=310). *** P<0.001. **E.** Plot of mitosis duration of control (wt), RNAi kinesin-14 (Kin-14), and double-RNAi kinesin-5 and kinesin-14 (Kin-5/Kin-14), separating cells with square spindles and diploid chromosome segregation from cells with abnormal spindles and aneuploid chromosome segregation. Control cells: diploid, 109 ± 45 min (n=64), aneuploidy, 267 ± 98 min (n=8); RNAi kinesin-14 (Kin-14): diploid, 93 ± 38 (n=7), aneuploid, 225 ± 190 min (n=309); double-RNAi kinesin-5/14 (Kin-5/Kin-14): diploid, 105 ± 26 (n=35), aneuploid, 304 ± 112 min (n=28). *** P<0.001.

## Description

We previously reported that the holocentric silkworm BmN4 cells have square-shaped metaphase spindles and relatively long mitosis duration compared to the monocentric human RPE1 cells with oval-shaped spindles and relatively short mitosis duration (Vanpoperinghe et al. 2021). Further, RNAi-mediated depletion of kinesin-5 produces the same phenotype in both organisms, creating monopolar spindle and cell death; and depletion of kinesin-14 significantly affected BmN4 cells, creating mal-shaped spindles, but had little effect on RPE1 cells (Vanpoperinghe et al. 2021). Here, we extended our analysis of depletion of kinesin-5 and kinesin-14 on spindle dynamics and chromosome segregation in BmN4 cells.

Long-term live cell-imaging, up to 20 hours, were performed on wild-type and RNAi-treated BmN4 cells stained with vital SPY-DNA and SPY-tubulin dyes to observe spindle and chromosome dynamics throughout the phases of mitosis. Control wild-type cells showed predominantly (91%) square-shaped metaphase spindle (Fig. 1A, 1C), with an average mitotic duration of 109 min (Fig. 1D). There were a small percentage of control cells (9%) which showed mal-shaped spindles, that led to unequal separation of chromosome, i.e., aneuploidy, at the end of mitosis (Fig. 1C), consistent with our previous report (Vanpoperinghe et al. 2021). We then performed RNAi-mediated depletion on kinesin-5 and kinesin-14 for 48 hours, and confirmed mRNA depletion up to 97% for kinesin-5 and 78% for kinesin-14 (Fig. 1B). Kinesin-5 depletion resulted in monopolar spindles and subsequent cell death in 97% of cells (Fig. 1A, 1C). Interestingly, we observed 3% of cells with mal-shaped spindles, resulting in aneuploidy, and 1 cell (<1%) with a normal square spindle and normal chromosome segregation (Fig. 1C). Kinesin-5 are known to organize the bipolar spindle in many monocentric organisms, including human. In contrast, holocentric organisms such as *C. elegans* can organize their spindle in the absence of kinesin-5 (Saunders et al. 2007). In this context, the regulation of the silkworm holocentric spindle has more similarity to the regulation of the human monocentric spindle.

Kinesin-14 depletion has diverse phenotypes. While 3% of cells showed the normal square-shaped spindles, 97% of cells showed mal-shaped spindles, which can be classified into two sub-phenotypes (Fig. 1A, 1C). Group Kin-14#1, representing 68% of the mal-shaped population, were able to complete mitosis but with abnormally long mitosis duration of 225 min (Fig. 1D). These spindles also exhibited aneuploidy, represented by the strong asymmetry in the DNA mass/area at telophase (Fig. 1D). Group Kin-14#2, representing 32% of the mal-shaped population, never completed mitosis during the 20 hours of observation (Fig. 1A). We speculate that the fate of group Kin-14#2 cells may be aneuploidy or apoptosis. Kinesin-14 is known to focus the microtubule minus ends at the spindle poles, and its inhibition, while having little effect on the metaphase spindle in normal human cells, causes catastrophic multipolar spindle defects in cancer cells with supernumerary centrosomes (Kleylein-Sohn et al. 2012; Kwon et al. 2008). In this context, the function of kinesin-14 in silkworm holocentric square spindle may be similar to its function in cancer cells.

It was previously reported that kinesin-5 and kinesin-14 are antagonistic motors involved in a force-balance mechanism to organize and maintain spindle bipolarity in monocentric budding and fission yeast – the simultaneous absence of both motors results in full rescue of spindle bipolarity and chromosome segregation (Saunders et al. 1997; Olmsted et al. 2014; Rincon et al. 2017: Yukawa et al. 2020). We performed simultaneous RNAi-mediated depletion for both kinesin-5 and kinesin-14. The double-depletion showed diverse phenotypes. Group Kin-5/14#1, representing 79% of cells, exhibited monopolar spindles and cell death (Fig. 1A, 1C). Group Kin-5/14#2, representing 11% of cells, exhibited mal-shaped spindles and aneuploidy (Fig. 1A, 1C). Interestingly, group Kin-5/14#3, representing 10% of cells, exhibited the wild-type-like square spindles and normal chromosome segregation (Fig. 1A, 1C). This indicates that in holocentric silkworm, kinesin-5 and kinesin-14 are partially antagonistic and can partially rescue the monopolar and mal-shaped spindle phenotypes. Throughout the experiments, we observed that mal-shaped spindles had prolonged mitosis duration as compared to square-shaped spindles; and that mal-shaped spindles led to aneuploidy while square-shaped spindle resulted in equal chromosome segregation (diploid). Our analysis of mitosis duration revealed that for all cells (control wild-type, Kin-14 depletion, and Kin-5/14 double-depletion), errors in chromosome segregation (aneuploidy) correlates strongly (p<0.001) with mal-shaped spindle and prolonged mitosis duration (Fig. 1E).

In summary, we report here that the square-shaped spindles of silkworm holocentric BmN4 cells are organized in part by kinesin-5 and kinesin-14, motors which are partially antagonistic, and that the proper shape of the spindle is highly correlated with mitosis duration and chromosome segregation errors. The differential significance of kinesin-14 for spindle regulation in silkworm cells compared to human cells could be associated with its peculiar square-shape morphology, thus further providing insights into the function of kinesin-14 in general. In addition, given the similar deleterious response to kinesin-14 depletion in silkworm cells and cancer cells with supermumerary centrosomes, we propose that BmN4 cells can also serve as a good model cell to study centrosome assembly and spindle architecture during malformation or malignant context.

## Methods

**Cells**

We used silkworm BmN4-SID1 (RRID:CVCL_Z091) cells (Mon et al., 2012). These immortalized cells are considered normal diploid cells, as they have proper number of chromosomes and do not exhibit transformed phenotypes. An added benefit is that they are easily amenable to RNAi-mediated depletion, enabling functional studies of protein-of-interest. Cell culture condition is well-established (Mon et al., 2012). Briefly, BmN4-SID1 cells were maintained in Sf-900 II SFM medium (GIBCO Cat# 10902-088) supplemented with 5% fetal calf serum (Biowest Cat# S181T-500), 1% penicillin-streptomycin (GIBCO Cat# 15140-122) and 1% L-glutamine (GIBCO Cat# 25030-024) in a 27°C incubator.

**RNAi**

RNA-interference for BmN4 cells has been established (Mon et al., 2012), utilizing the ability of BmN4-SID1 cells to soak up dsRNA in the medium to perform RNA-interference. Briefly, total mRNA was extracted from BmN4 cells using the TRIzol RNA Isolation Reagents Kit (Thermo Fisher Cat# 15596026). Then total cDNA was reversed transcribed from the mRNA pool using the SuperScript III Reverse Transcriptase Kit (Thermo Fisher Cat# 18080093). Then ~300 bp of dsRNA targeting silkworm kinesin-5 and kinesin-14 were generated from the cDNA pool flanked by T7 promoter sequences on both sides using the MAXIscript T7 Transcription Kit (Thermo Fisher Scientific, Cat# AM1312). We treated BmN4-SID1 cells with 20 nM of dsRNA 48 hr prior to imaging. A subset of the treated-cells were used to determine the level of mRNA depletion by semi-quantitative PCR and gel-electrophoresis.

**Live-cell imaging**

The cells were cultured in a 2 mL FluoroDish (World Precision Instruments, Cat# FD35-100). One hour prior to imaging, SPY-DNA-555nm (Spirochrome Cat# SC201) and SPY-tubulin-650nm (Spirochrome Cat# SC503) dyes were added to the medium at a 10,000X dilution. Cells were imaged using the spinning disk confocal microscope. Briefly, the Nikon Eclipse Ti-E perfect focus inverted microscope, with 40X/1.3 N.A. Plan Apo oil immersion objective lens and Mad City Piezo stepper stage, coupled to the Yokogawa CSU-X1 spinning disk confocal unit, the Photometrics Cascade EM-CCD camera, the Gataca Systems laser unit with 561 nm (100 mW) and 642 nm (100 mW) lines, controlled by Molecular Devices software MetaMorph 7.8, enclosed within a thermal box to keep stable temperatures of 27°C. Movies were made with the following parameters: laser power 5%, EM-gain 300, Bin 1X, exposure time 100-200 ms, 13 optical z-sections, 2 µm spacing per 3D stack, 5 min time interval between stacks, 6-20 hr movies.

## Reagents

*Bombyx mori* primers to generate T7 template for kinesin-5 (SilkDB 3.0: BMSK0004112) dsRNA:

sense: GCTGGCTACAATTGCACTGT

antisense: TCATGAGTGTCGAAGCCACT

*Bombyx mori* primers to generate T7 template for kinesin-14 (SilkDB 3.0: BMSK0005503) dsRNA:

sense: TGCTGCACCTCGGATTAAGA

antisense: CCTCTAGAAGCTTTGTCTTACGT
